# Neurosurgical Emergencies in Sports Neurology

**DOI:** 10.1007/s11916-016-0586-4

**Published:** 2016-08-17

**Authors:** Vin Shen Ban, James A. Botros, Christopher J. Madden, H. Hunt Batjer

**Affiliations:** Department of Neurological Surgery, University of Texas Southwestern Medical Center, 5323 Harry Hines Blvd, Dallas, TX 75390-8855 USA

**Keywords:** Neurosurgical emergencies, Sports-related TBI, Traumatic brain injury, Second impact syndrome

## Abstract

**Purpose of Review:**

Athletic neurosurgical emergencies are injuries that can lead to mortality or significant morbidity and require immediate recognition and treatment. This review article discusses the epidemiology of sports-related traumatic brain injury (TBI) with an attempt to quantify the incidence of neurosurgical emergencies in sports. Emergencies such as intracranial hemorrhage, second impact syndrome, vascular injuries, and seizures are discussed.

**Recent Findings:**

The incidence of sports-related TBI presenting to level I or II trauma centers in the USA is about 10 in 100,000 population per year. About 14 % of the adult sports-related TBIs and 13 % of the pediatric sports-related TBIs were moderate or severe in nature. Patients presenting with headache and neck pain should prompt further investigation for cervical spine and vascular injuries. CT angiography is becoming the modality of choice to screen for blunt cerebrovascular injuries. The treatment of these injuries remains controversial.

**Summary:**

High-quality evidence in sports-related TBI is lacking. Further research is required to help guide management of this increasingly prevalent condition. The role of prevention and education should also not be underestimated.

## Introduction

Athletic injuries are common, but injuries involving the brain or spinal cord are less so. Concussions, a subset of mild traumatic brain injuries (mTBI), are the most common type of head injury and have received significant recent media attention [1•, 2]. While there can be significant sequelae of a concussion, management generally involves removal from play and supportive care while symptoms are present. The current article focuses on those injuries of the brain and spinal cord that represent a significant immediate risk of deterioration and whose recognition and treatment may prevent progressive disability or death. Neurological deficits presenting during an athletic contest should warrant prompt medical attention to evaluate for these conditions that may need immediate care.

## Epidemiology

In the USA, TBI was a contributing factor in 30.5 % of injury-related deaths [3]. According to the CDC, about 1.7 million TBIs present to emergency departments annually. The proportion of TBIs related to athletic injuries is unclear. It has been estimated that 3.8 million sports-related TBIs occur annually, including those who did not seek medical care [4]. It is likely that the moderate and severe TBIs (as determined by Glasgow Coma Scale [GCS] of 9–12 and 3–8, respectively) were more likely to present to emergency departments compared to the mild TBIs (GCS 13–15).

Recently, a group from UCSF has utilized data from the National Sample Program of the National Trauma Data Bank to describe the incidence of pediatric and adult sports-related traumatic brain injury in US Trauma Centers [5•, 6•]. They estimated that 18,310 adult and 11,614 pediatric TBIs present to level I or level II trauma centers in the USA each year. This represents an incidence of almost 10 in 100,000 athletic-associated TBIs per year in the USA. About 14 % of the adult sports-related TBIs and 13 % of the pediatric sports-related TBIs were moderate or severe in nature. In the adult population, mortality after sports-related TBI was 3.1 %. Of those sports-related deaths, roller sports (4.1 %) and aquatic sports (7.7 %) were the activities associated with the highest death rates. In children, the mortality was 0.8 % of sports-related TBI. The authors found that the severity of head and extracranial injuries predicted prolonged hospital and ICU stays, medical complications, failure to discharge home, and death, with age and hypotension being additional predictors for the adult population but not for children [5•, 6•].

In Nova Scotia where there was an estimated pediatric population of 200,000, the Nova Scotia Trauma Registry recorded 107 pediatric major trauma cases related to sports over a 13-year period [7•]. About 52 % were classified as severe TBIs (Abbreviated Injury Scale [AIS]-90 head score ≥3). This translates into an incidence of approximately 2.2 severe TBIs per 100,000 populations per year. Cycling represented 55 % of the major sports-related trauma, and 54 % of the cycling injuries were severe TBIs.

## Sports-Specific Injuries

Sports injuries can be broadly classified according to mechanism (penetrating versus blunt injuries) and structure (vascular versus parenchymal injuries). The mechanisms of injury leading to neurosurgical emergencies are often sports-specific, as demonstrated by high-profile cases reported in the media (Table 1).

**Table 1:** Non-exhaustive list of high-profile sports-related neurosurgical emergencies based on media reports

Case	Year	Sports	Injury	Outcome
Vladimir Smirnov	1982	Fencing (foil)	Penetrating brain injury through orbit	Death
Clint Malarchuk	1989	Ice hockey	Severed carotid artery and internal jugular vein	Good recovery
Christopher Reeve	1995	Equestrian (cross country)	C1-2 fracture, SCI	Severely disabled
Zachary Lystedt	2006	Football	Bilateral subdural hematoma	Severely disabled
Richard Zednik	2008	Ice hockey	Lacerated external carotid artery	Good recovery
Natasha Richardson	2009	Skiing (off-piste)	Epidural hematoma	Death
Michael Schumacher	2013	Skiing (off-piste)	TBI, exact injuries unknown	Severely disabled

## Intracranial Hemorrhage

Sports injuries resulting in intracranial hemorrhage such as acute subdural hematoma (ASDH), epidural hematoma (EDH), and intracerebral hemorrhage (ICH) typically occur through sports with the potential for high-energy collisions such as motorsports (formula racing, rallying), boardsports (skateboarding, snowboarding), and skiing. Patients on anticoagulation are particularly challenging to manage. The topic of anticoagulation in the neurosurgical patient has recently been extensively reviewed [8].

A recent systematic review and meta-analysis of cohort studies found that in ASDH patients (of all causes, including sports) who presented with bilateral fixed and dilated pupils, the mortality after undergoing surgery was 66 % while only 6.6 % achieved a favorable Glasgow Outcome Scale (GOS) score [9]. In the EDH cohort, the mortality was found to be almost 30 %, and 54 % achieved a favorable GOS. While age has been shown to be a predictive factor for poorer outcomes [10], “age bias” in terms of the patient management in certain regions may have confounded this issue [11].

## Second Impact Syndrome

The concept that a second impact leading to a catastrophic brain injury after a concussion was thought to be first described by Schneider in 1973 [12, 13]. The term “second impact” was then used by Saunders and Harbaugh in their 1984 report of a case of a 19-year-old college football player who developed brain swelling leading to death after playing football with no apparent trauma [13]. It became clear that this patient had received a blow to the head 4 days prior and was still symptomatic when he resumed contact play. The autopsy findings led the authors to speculate that the catastrophic brain edema was a result of reduced brain compliance from the recent head injury. Nonetheless, the existence of second impact syndrome (SIS) has previously been challenged [14].

A recent systematic review investigated the cases published in the literature to identify unique criteria for assigning the diagnosis of SIS [15]. Of the eight articles included in their final analysis, the authors found that young adults or adolescents, brain swelling, herniation, and acute subdural hematoma were the common features in most cases. However, the authors concluded that the development of a unique International Classification of Diseases (ICD) diagnosis of SIS was not supported by the evidence available in the literature, citing the overlapping symptoms with various other conditions, the lack of an established timeline between the head impacts, and the mixed results of the presence of imaging findings as reasons. Despite the lack of convincing evidence, an approach of ensuring the resolution of symptoms prior to return to play is advocated in current return to play guidelines [1•].

## Vascular Injuries

There is no epidemiological literature on vascular injuries that is specific to the athletic population. Vascular injury will be a rare injury in the athletic setting but in the trauma literature, blunt cervical vascular injuries (BCVI) can occur in the setting of high-energy impacts and can have the potential to lead to embolic stroke and death. These injuries are typically caused by impingement of the vessel by a bony surface, by stretching or tearing during vigorous rotation of the neck, or by spinal or skull base fractures lacerating the vessel. The resulting intimal tear predisposes to thrombus formation that can lead to complete vessel occlusion or distal emboli. Pseudoaneurysms or complete transections are significantly less common types of BCVI.

Typically, symptomatic patients present with headaches, neck pain, Horner’s syndrome, or stroke. As headaches and neck pain are common in the TBI population, a high index of suspicion for a vascular injury should be present. A review of headaches in intracranial and cervical artery dissections was undertaken in a previous issue of this journal [16]. Patients presenting with these symptoms after a sports-related TBI should prompt investigation for cervical spine fracture and BCVI. Cervical spine fracture has previously been shown to be an independent predictor of blunt vertebral artery injury [17•, 18•]. Other high-risk features that should warrant screening for BCVI have been defined in the Denver criteria [17•, 19•, 20, 21]. These include basilar skull fractures, Lefort II and III fractures, and penetrating injuries. The Denver group has also introduced a grading scale for BCVIs which correlated with the risk of stroke—grade I (luminal irregularity or dissection with <25 % luminal stenosis), grade II (dissection or intramural hematoma with ≥25 % luminal stenosis, intraluminal thrombus, or raised intimal flap), grade III (pseudoaneurysm), grade IV (occlusion), and grade V (transection) [19•].

Recent literature suggests that cerebral infarctions may occur prior to presentation or shortly after arrival [22–27]. Cothren and colleagues argued for comprehensive screening and early diagnosis based on their findings of stroke in 21 % of their BCVI patients [28], although that percentage varies with other series [22–26, 29]. Out of their cohort of 301 patients with BCVIs, 3.7 % were due to snowboarding, and 1.7 % were due to bicycling. While the authors acknowledged the subgroup of 11 patients who presented with neurologic symptoms of stroke within 1–2 h, they have noted that another subgroup of 34 patients also exist where these patients remained asymptomatic in the first 2 h, but subsequently developed stroke with a mean time of 75 h.

In terms of screening modality, digital subtraction angiography (DSA) is considered to be the gold standard diagnostic test, although computed tomography angiography (CTA) has been shown to have a good sensitivity as a screening modality and is used in many institutions [30••, 31].

The most appropriate treatment of vascular injury remains a topic of debate. Anticoagulant and antiplatelet agents carry the risk of hemorrhagic complications especially in a trauma cohort but may lessen the risk of developing a thrombus and an ischemic complication [32]. Endovascular intervention may have a role in the treatment of pseudoaneurysms, but concerns have been raised about the procedure-related complications [33]. Open surgical repair is only rarely warranted. Guidelines for the management of BCVI have been published in the trauma literature [34••, 35••].

## Seizures

Seizure is perhaps one of the most dramatic manifestations of acute sports-related head injury. While rare, convulsive episodes following mild traumatic brain injury represent an important diagnostic dilemma. It is estimated that up to one in 70 patients with concussion experience a concussive convulsion immediately following impact [36]. This may include an immediate tonic phase followed by a clonic or myoclonic phase lasting several minutes. Post-ictal state is either absent or very brief. Previously referred to as impact seizures, these episodes are more frequently referred to as concussive convulsion to emphasize the non-epileptic nature of these events [37]. Although the exact pathophysiology of these events is unclear, the dominant theory incorporates the concept of rapid, non-discriminate release of excitatory neurotransmitters at the time of impact, followed shortly by spreading depression. This is believed to result in loss of cortical inhibition, which in turn results in transient functional decerebration, which manifests as concussive convulsion [36, 38•]. Studies have demonstrated that there is no correlation between the presence of concussive convulsion and adverse clinical or radiographic outcome, and there is no increased risk of post-traumatic epilepsy. Therefore, no specific treatment is necessary beyond immediate supportive care and airway protection. There is no indication for anticonvulsant therapy [39]. Due to the benign nature of concussive convulsion, it is not considered a concussion modifier for the purposes of classifying concussion severity and guiding return to play or other post-concussion counseling [1•].

Seizures may also represent a manifestation of an underlying parenchymal brain injury however. In the pre-hospital setting, prolonged seizure activity may indicate a more severe head injury, potentially with structural brain injury such as intracranial contusion or extra-axial hematoma. Prolonged seizure activity warrants hospital transfer and cranial imaging.

Post-traumatic seizures are typically divided into early seizures, occurring within 1 week of injury, and late seizures, occurring more than 1 week after injury. Estimated risk of post-traumatic seizures varies from 2 to 25 % and risk increases with head injury severity [40]. Prolonged unconsciousness or post-traumatic amnesia, particularly beyond 24 h, are both recognized as risk factors [41]. Penetrating injury has the strongest correlation with post-traumatic seizures, with estimates up to 53 %. Patients with severe or penetrating head injury should receive seizure prophylaxis for at least 7 days. It should be recognized, however, that this decreases the risk of early seizures but does not significantly reduce the risk of late seizures or post-traumatic epilepsy [40, 41].

## Neck Injuries, Penetrating Injuries, CSF Leak, Incidental Findings

Cervical spine fractures are a concern in sports injuries. As discussed in the section on vascular injuries above, the sudden onset of severe neck pain should raise suspicion for vascular injuries, cervical spine fractures, and ligamentous injuries. These patients should be stabilized according to the advanced trauma life support (ATLS) protocol immediately, with specific emphasis on head and neck immobilization. They should be removed from the field of play and transferred to the emergency room as soon as possible. Occasionally, blows to the head have concomitant transfer of forces to the cervical spine that may present with spinal concussion-like symptoms. Due to the potential for unstable cervical spine injuries, any patient with a neurological deficit, even transient, should be treated as if they also have a cervical spine injury until proven otherwise [42, 43].

Penetrating cranial or spinal injuries need immediate hospital transfer for definitive care. In general, the penetrating object should not be removed but should be stabilized for transport. Cerebrospinal fluid (CSF) leak (otorrhea or rhinorrhea) may be associated with penetrating injury or with fractures through the skull base. An athlete with drainage from the ear or nose consistent with a CSF leak will require immediate medical evaluation due to the high risk of meningitis.

A proportion of sports injuries may reveal incidental findings in the brain and spinal cord, or trigger the symptom onset of these lesions. For example, an AVM might bleed after a blow to the head. An athlete with a type I Chiari malformation may experience Lhermitte’s sign (masquerading as “stingers”) or may be quadriplegic from head trauma. Despite the rare occurrence of these scenarios, there should be a low threshold for transfer to an emergency room for further evaluation and management.

## Concluding remarks

As we begin to further our understanding of TBIs, the role of prevention should also be acknowledged. One of the focuses of this area is on the use of helmets. Rapid design and technological improvements have given rise to a range of measurements to evaluate the protective effect of these helmets [44]. How these translate into the prevention of head injuries remains to be seen. The role of education, rule changes, and legislation should also not be underestimated in the quest to prevent sports-related TBIs [45].

While neurosurgical emergencies in sports can have devastating consequences, the relative rarity of these emergencies in the athletic setting has resulted in few treatment recommendations specific to athletics. Prospective longitudinal population studies and TBI injury registries should be encouraged. In the meantime, it is reasonable to extrapolate principles from the non-athletic trauma literature to support decisions regarding diagnosis and treatment. Any athlete exhibiting neurological symptoms on the field or after an athletic contest requires an expeditious medical evaluation.

## References

[1.•] McCrory P, Meeuwisse WH, Aubry M, Cantu B, Dvorak J, Echemendia RJ, et al. Consensus statement on concussion in sport: the 4th International Conference on Concussion in Sport held in Zurich, November 2012. J Am Coll Surg. 2013;216(5):e55–71. **The latest international consensus statement on sports concussion. This consensus statement highlights the lack of high quality evidence for the diagnosis and management of sports concussion. An updated concussion statement is due soon**. 10.1016/j.jamcollsurg.2013.02.020 23582174

[2] Ban VS, Madden CJ, Bailes JE, Hunt Batjer H, Lonser RR (2016). The science and questions surrounding chronic traumatic encephalopathy. Neurosurg. Focus.

[3] Faul M, Xu L, Wald MM, VG C. Traumatic brain injury in the United States: emergency department visits, hospitalizations, and deaths, 2002-2006. Centers for Disease Control and Prevention, National Center for Injury Prevention and Control. Atlanta, GA; 2010.

[4] Langlois JA, Rutland-Brown W, Wald MM (2006). The epidemiology and impact of traumatic brain injury: a brief overview. J. Head Trauma Rehabil..

[5.•] Yue JK, Winkler EA, Burke JF, Chan AK, Dhall SS, Berger MS (2016). Pediatric sports-related traumatic brain injury in United States trauma centers. Neurosurg. Focus.

[6.•] Winkler EA, Yue JK, Burke JF, Chan AK, Dhall SS, Berger MS (2016). Adult sports-related traumatic brain injury in United States trauma centers. Neurosurg. Focus.

[7.•] Green RS, Butler MB, Kureshi N, Erdogan M. A retrospective evaluation of pediatric major trauma related to sport and recreational activities in Nova Scotia. CJEM. 2016;18(2):106–11. **Another trauma registry study, with a focus on the pediatric population in one of the most densely populated provinces in Canada**10.1017/cem.2015.69 26212386

[8] Le Roux P, Pollack CV, Milan M, Schaefer A (2014). Race against the clock: overcoming challenges in the management of anticoagulant-associated intracerebral hemorrhage. J. Neurosurg..

[9] Scotter J, Hendrickson S, Marcus HJ, Wilson MH (2015). Prognosis of patients with bilateral fixed dilated pupils secondary to traumatic extradural or subdural haematoma who undergo surgery: a systematic review and meta-analysis. Emerg. Med. J..

[10] Susman M, DiRusso SM, Sullivan T, Risucci D, Nealon P, Cuff S (2002). Traumatic brain injury in the elderly: increased mortality and worse functional outcome at discharge despite lower injury severity. J. Trauma.

[11] Kirkman MA, Jenks T, Bouamra O, Edwards A, Yates D, Wilson MH (2013). Increased mortality associated with cerebral contusions following trauma in the elderly: bad patients or bad management?. J. Neurotrauma.

[12] Schneider R (1973). Head and neck injuries in football: mechanisms, treatment, and prevention.

[13] Saunders RL, Harbaugh RE (1984). The second impact in catastrophic contact-sports head trauma. JAMA.

[14] McCrory P (2001). Does second impact syndrome exist?. Clin. J. Sport Med..

[15] Hebert O, Schlueter K, Hornsby M, Van Gorder S, Snodgrass S, Cook C. The diagnostic credibility of second impact syndrome: a systematic literature review. J Sci Med Sport. 2016. doi:10.1016/j.jsams.2015.12.517. 10.1016/j.jsams.2015.12.517 26795449

[16] Sheikh HU (2016). Headache in intracranial and cervical artery dissections. Curr Pain Headache Rep.

[17.•] Biffl WL, Moore EE, Offner PJ, Brega KE, Franciose RJ, Elliott JP, et al. Optimizing screening for blunt cerebrovascular injuries. Am J Surg. 1999;178(6):517–22. **The modified Denver criteria for BCVI screening. High risk factors for blunt carotid injuries include GCS ≤ 6, petrous fracture, diffuse axonal injury, and LeFort II or III fractures. Cervical spine fractures was identified as a high risk factor for blunt vertebral artery injury**. 10.1016/s0002-9610(99)00245-7 10670864

[18.•] Franz RW, Willette PA, Wood MJ, Wright ML, Hartman JF. A systematic review and meta-analysis of diagnostic screening criteria for blunt cerebrovascular injuries. J Am Coll Surg. 2012;214(3):313–27. **A recent systematic review and meta-analysis of diagnostic screening criteria for blunt cerebrovascular injuries. Cervical and thoracic spine injuries were found to be significant predictors of BCVI**. 10.1016/j.jamcollsurg.2011.11.012 22244206

[19.•] Biffl WL, Moore EE, Offner PJ, Brega KE, Franciose RJ, Burch JM. Blunt carotid arterial injuries: implications of a new grading scale. J Trauma. 1999;47(5):845–53. **The article on the BCVI grading scale by the Denver group**. 10.1097/00005373-199911000-00004 10568710

[20] Biffl WL, Moore EE, Elliott JP, Ray C, Offner PJ, Franciose RJ (2000). The devastating potential of blunt vertebral arterial injuries. Ann. Surg..

[21] Biffl WL, Moore EE, Offner PJ, Burch JM (2001). Blunt carotid and vertebral arterial injuries. World J. Surg..

[22] Scott WW, Sharp S, Figueroa SA, Madden CJ, Rickert KL (2014). Clinical and radiological outcomes following traumatic grade 1 and 2 vertebral artery injuries: a 10-year retrospective analysis from a level 1 trauma center. J. Neurosurg..

[23] Scott WW, Sharp S, Figueroa SA, Eastman AL, Hatchette CV, Madden CJ (2015). Clinical and radiological outcomes following traumatic grade 3 and 4 vertebral artery injuries: a 10-year retrospective analysis from a level I trauma center. The Parkland Carotid and vertebral artery injury survey. J. Neurosurg..

[24] Scott WW, Sharp S, Figueroa SA, Eastman AL, Hatchette CV, Madden CJ (2015). Clinical and radiographic outcomes following traumatic grade 1 and 2 carotid artery injuries: a 10-year retrospective analysis from a level I trauma center. The Parkland Carotid and vertebral artery injury survey. J. Neurosurg..

[25] Scott WW, Sharp S, Figueroa SA, Eastman AL, Hatchette CV, Madden CJ (2015). Clinical and radiographic outcomes following traumatic grade 3 and 4 carotid artery injuries: a 10-year retrospective analysis from a level 1 trauma center. The Parkland Carotid and vertebral artery injury survey. J. Neurosurg..

[26] Griessenauer CJ, Fleming JB, Richards BF, Cava LP, Cure JK, Younan DS (2013). Timing and mechanism of ischemic stroke due to extracranial blunt traumatic cerebrovascular injury. J. Neurosurg..

[27] Mayberry JC, Brown CV, Mullins RJ, Velmahos GC. Blunt carotid artery injury: the futility of aggressive screening and diagnosis. Arch Surg. 2004;139(6):609–12. discussion 12-3. 10.1001/archsurg.139.6.609 15197086

[28] Cothren CC, Biffl WL, Moore EE, Kashuk JL, Johnson JL (2009). Treatment for blunt cerebrovascular injuries: equivalence of anticoagulation and antiplatelet agents. Arch Surg.

[29] Stein DM, Boswell S, Sliker CW, Lui FY, Scalea TM (2009). Blunt cerebrovascular injuries: does treatment always matter?. J. Trauma.

[30.••] Roberts DJ, Chaubey VP, Zygun DA, Lorenzetti D, Faris PD, Ball CG, et al. Diagnostic accuracy of computed tomographic angiography for blunt cerebrovascular injury detection in trauma patients: a systematic review and meta-analysis. Ann Surg. 2013;257(4):621–32. Epub 2013/03/09. **A systematic review and meta-analysis of eight studies to quantify the diagnostic accuracy of CTA compared to catheter angiogram for detecting BCVI. The authors found that CTA has high specificity but low sensitivity for this purpose**. 10.1097/SLA.0b013e318288c514 23470509

[31] Shahan CP, Magnotti LJ, Stickley SM, Weinberg JA, Hendrick LE, Uhlmann RA, et al. A safe and effective management strategy for blunt cerebrovascular injury: avoiding unnecessary anticoagulation and eliminating stroke. J Trauma Acute Care Surg. 2016;80(6):915–22. 10.1097/TA.0000000000001041 27015579

[32] Shahan CP, Magnotti LJ, McBeth PB, Weinberg JA, Croce MA, Fabian TC. Early antithrombotic therapy is safe and effective in patients with blunt cerebrovascular injury and solid organ injury or traumatic brain injury. J Trauma Acute Care Surg. 2016;81(1):173–7. 10.1097/TA.0000000000001058 27027559

[33] Cothren CC, Moore EE, Ray CE, Ciesla DJ, Johnson JL, Moore JB (2005). Carotid artery stents for blunt cerebrovascular injury: risks exceed benefits. Arch Surg.

[34.••] Bromberg WJ, Collier BC, Diebel LN, Dwyer KM, Holevar MR, Jacobs DG (2010). Blunt cerebrovascular injury practice management guidelines: the Eastern Association for the Surgery of Trauma. J. Trauma.

[35.••] Biffl WL, Cothren CC, Moore EE, Kozar R, Cocanour C, Davis JW (2009). Western Trauma Association critical decisions in trauma: screening for and treatment of blunt cerebrovascular injuries. J. Trauma.

[36] McCrory PR, Berkovic SF (1998). Concussive convulsions. Incidence in sport and treatment recommendations. Sports Med..

[37] Perron AD, Brady WJ, Huff JS (2001). Concussive convulsions: emergency department assessment and management of a frequently misunderstood entity. Acad Emerg Med.

[38.•] Giza CC, Hovda DA (2014). The new neurometabolic cascade of concussion. Neurosurgery.

[39] Nass RD, Elger CE, Fink GR, Burghaus L. Concussive convulsions: seizure or no seizure? Fortschr Neurol Psychiatr. 2011;79(11):655–9. Kommotionelle Konvulsionen: Epileptischer Anfall oder nicht?. 10.1055/s-0031-1281688 22002819

[40] Garga N, Lowenstein DH (2006). Posttraumatic epilepsy: a major problem in desperate need of major advances. Epilepsy Curr.

[41] Ferguson PL, Smith GM, Wannamaker BB, Thurman DJ, Pickelsimer EE, Selassie AW (2010). A population-based study of risk of epilepsy after hospitalization for traumatic brain injury. Epilepsia.

[42] Leddy JJ, Baker JG, Merchant A, Picano J, Gaile D, Matuszak J (2015). Brain or strain? Symptoms alone do not distinguish physiologic concussion from cervical/vestibular injury. Clin. J. Sport Med..

[43] Jagannathan J, Dumont AS, Prevedello DM, Shaffrey CI, Jane Jr JA. Cervical spine injuries in pediatric athletes: mechanisms and management. Neurosurg Focus. 2006;21(4). 10.3171/foc.2006.21.4.7 17112196

[44] Hoshizaki TB, Post A, Oeur RA, Brien SE (2014). Current and future concepts in helmet and sports injury prevention. Neurosurgery..

[45] Ellenbogen RG, Berger MS, Batjer HH (2010). The National Football League and concussion: leading a culture change in contact sports. World Neurosurgery.

